# Regionalizing healthcare: a vision for transforming Lebanon into a regional academic hub

**DOI:** 10.1186/1472-6963-10-167

**Published:** 2010-06-16

**Authors:** Kamal F Badr, Elie A Akl

**Affiliations:** 1 Dean's office, Lebanese American University, Beirut, Lebanon; 2 Department of Medicine, State University of New York at Buffalo, Buffalo, NY, USA

## Abstract

**Background:**

Lebanon suffers from a large scale emigration of physicians coupled with an oversaturation of the physician job market. Lebanon is currently witnessing an expansion of its medical education capacity with the establishment of new private medical schools, raising the fears of a worsening market oversaturation.

**Discussion:**

The neighboring Arabian Gulf countries are suffering from a serious shortage of clinicians and academicians. In spite of their enormous investments in educational, clinical and research collaborative initiatives with some of the most renowned North American medical schools and institutions, their ability to recruit and retain highly qualified clinicians and academicians remains a major challenge. Lebanese universities have the opportunity to establish triangular collaborations with the Gulf regional medical centers and their North American partners. They could achieve this goal by tapping into the globalized and high quality Lebanese physician workforce and consequently regionalize healthcare delivery in the Middle East.

**Summary:**

By recruiting its globalized and high quality physician workforce to establish collaborations with the Gulf regional, Lebanon could become a regional "academic hub".

## Background

**Figure 1:**
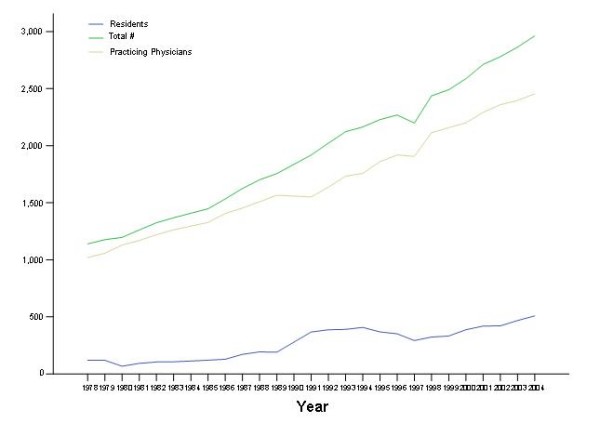
**Time trends (1978-2004) of the number of Lebanese medical graduates in the US **[2].

In 2004, Lebanon had the highest physician emigration factor of all the countries in the Middle East and North Africa, and the 7^th ^highest in the world [1]. The total number of Lebanese medical graduates (LMGs) practicing in the Unites States of America (USA) today is estimated at close to three thousand, and this number been increasing steadily (Figure 1) [2]. Indeed, in 2004, about 40% of those who had graduated from Lebanese medical schools in the preceding 25 years were active physicians in the US [2].

**Figure 2:**
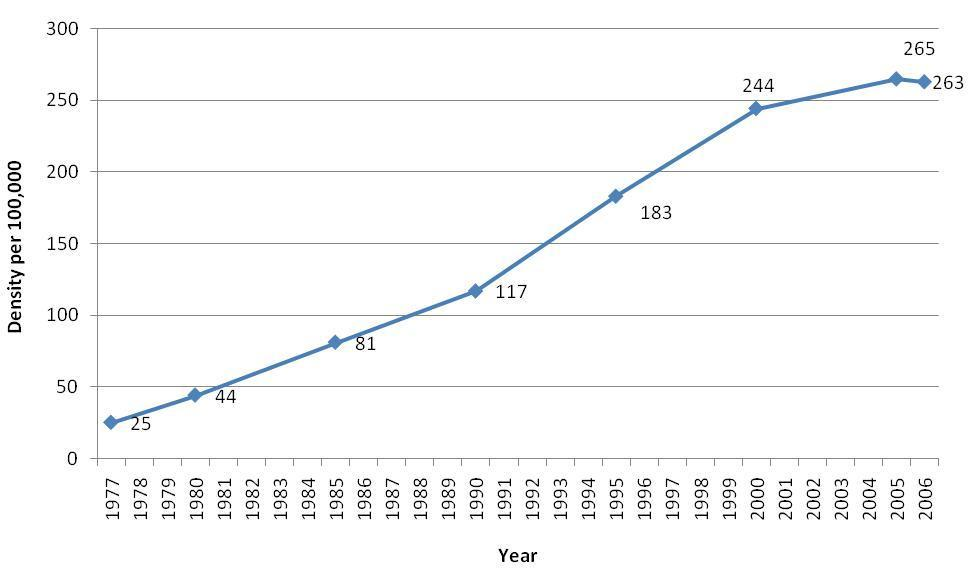
**Physician density in Lebanon over the 1977-2006 period **[5].

A recent study found that the physician density in Lebanon grew by a multiple of 11 over the last 30 years reaching 263 per 100,000 in 2006 (Figure 2) [3]. This number approximates the physician density in the US (293 per 100,000) but exceeds that of many countries such as Canada and the United Kingdom [1]. A perceived oversaturation of the Lebanese physician job market has encouraged medical students to travel abroad [4]. A survey study found that about 96% of students of Lebanese medical schools intend to migrate for post graduate training with a minority intending to return directly to Lebanon after finishing training abroad [5].

Lebanon is currently witnessing an expansion of its medical education capacity with the establishment of new private medical schools [Additional file 1]. This expansion does not appear to be related to a governmental policy, nor is it in response to the needs of the country as such, as the physician density in Lebanon exceeds most countries'. Rather, these are privately funded initiatives, many by religious orders, which do not receive public support. One of the objectives of these schools is to equip graduates with an international level of education enabling them to enter the global market (unpublished data). However, many worry that such policy is forcing the emigration of Lebanese physicians. This paper explores how a vision of regionalizing healthcare can turn threats into opportunities. This vision is built on 2 notions: a high quality globalized Lebanese physician workforce and a regional shortage of clinicians and academicians.

## Discussion

### A high quality globalized Lebanese physician workforce

LMGs have higher likelihoods of being certified by medical specialty boards (compared to other graduates in the USA), of engaging in research, of being affiliated with universities and of pursuing academic careers [2]. In many cases, LMGs are leaders of academic departments, research laboratories and institutes in distinguished American medical centers and institutions.

### A regional shortage of clinicians and academicians

In contrast to the oversupply of the Lebanese physician workforce, countries in the Arabian Gulf region are facing a shortage of physicians. The Arabian Gulf region has successfully attracted foreign workers who now constitute as much as 80% of the population of some of these countries [6]. Sustained population growth coupled with an accelerated rise in chronic disease has resulted in a relative shortage of hospital beds and of doctors in certain specialties and sub-specialties [7]. Questions have been raised about the ability of Gulf governments to continue providing relatively high standards of healthcare [8].

Countries such as Oman and Saudi Arabia have successfully recruited foreign health workers to fill critical gaps, as an interim strategy [9]. Currently, hospital construction plans in the Gulf region are estimated to cost $14 billion [6]. In parallel, and over the past few years, North American medical institutions such as Weill-Cornell, The Cleveland Clinic, Johns Hopkins, and Harvard have been involved in the development of Middle East based clinical care centers, programs in medical education and continuing medical education, and clinical and basic medical research centers [10-13].

Despite the enormous investments by these prestigious American medical institutions, these efforts have apparently not met expectations, as the result of two major challenges. The first challenge is the dearth of qualified academic partners and/or 'homes' for their North American counterparts, as there are few local universities in the Gulf region with deep histories and traditions in clinical care, medical education or medical research. The second challenge is the difficulty in recruiting and retaining sufficient numbers of high quality medical and paramedical clinicians or faculty members to support the development and sustain the growth of robust clinical care centers, medical education programs, or research centers. It is particularly difficult to convince North American physicians, nurses, trainers, and technical staff to spend extended periods of time outside their home countries, especially given the distinct cultural and social norms of the Middle-East. This has inevitably led to difficulty in attracting students and post-graduate trainees to these centers. Furthermore, highly regarded bodies of accreditations of post-graduate training programs and medical schools in the Gulf region (similar to the Liaison Committee on Medical Education (LCME) and the Accreditation Council for Graduate Medical Education (ACGME)) do not exist.

### Establishing the vision, seizing the opportunity

The challenges North American medical schools are facing in attempting to establish partnerships in the region present an excellent opportunity for Lebanese medical academic institutions. Indeed, these institutions can play a constructive role in facilitating the success of the North American initiatives, while positioning themselves to benefit greatly from their presence. They could potentially attain regional pre-eminence by pursuing the strategic objective of establishing triangular collaborations with the Gulf regional medical centers and their North American partners.

Lebanese academic medical institutions, particularly those that follow the American model of medical education, training and practice, should form a regional "academic hub" or "anchor" for collaborative efforts with regional medical centers. These institutions should therefore establish strong clinical networks, high quality and internationally accredited educational programs, and robust basic science and clinical research units. As regional medical centers and medical schools sprout (with or without American academic partnerships), their need for high quality clinicians, teachers, researchers, physician-scientists, post-graduate trainees, paramedical personnel, nurses, and other categories of health care workers will increase progressively over the next few decades. Lebanese medical institutions following the American medicine model should therefore organize and position themselves as a dynamic base of high-quality human resources to propel the development of regional centers of excellence in research and clinical medicine.

Practical steps to attain the status of a regional "academic hub" could include:

• Establishing collaborative efforts with regional medical centers

• Determining and monitoring the specific regional needs for physicians (clinicians, educators, researchers and executive)

• Defining and characterizing the group of Lebanese physicians in the US, the potential source of recruitment

• Establishing the clinical, and academic infrastructure to repatriate physicians

• Establishing the business infrastructures to repatriate physicians;

• The practice model would have the physicians (and their families) based in Lebanon while providing services in the Arabian Gulf region as locum tenens

Given the right offers, many Lebanese medical and other health profession graduates are likely to elect to return to the homeland, and reuniting them and their children with their country and their families. Needless to say, the benefits to Lebanon in human, educational and economic terms emanating from a large-scale return of its health professionals will be a much-needed boost. Lebanese Medicine is as much a national treasure as many oil wells and its time has come.

### Challenges

There are a number of challenges to the implementation of this proposal. First, the proposal depends on its acceptability among policy makers both in the Arabian Gulf region and in Lebanon. While data is lacking for the former group, a recent qualitative study conducted among deans of Lebanese medical schools, heads of professional organizations as well as government officials suggests an enthusiasm for partnerships and involvement at both the regional and global levels (unpublished data). Second, there is a question about the ability to attract LMGs to work in the Gulf region. In a recent survey, we found that about a third of LMGs in the US are willing to locate to the Arabian Gulf region (unpublished data). More than half are willing to relocate to Lebanon as a base for locum tenens jobs in the Arabian Gulf region. However, whether willingness translates into actual actions remains a question.

## Summary

• Lebanon has a very high physician emigration factor and its medical graduates form a high quality globalized physician workforce

• The neighboring Arabian Gulf countries are suffering from a serious shortage of clinicians and academicians that has been very challenging to reverse.

• Lebanese universities have an opportunity to tap into the globalized and high quality Lebanese physician workforce to regionalize healthcare delivery in the Middle East.

• Pursuing this strategic objective can make Lebanon the regional "academic hub".

## Abbreviations

ACGME: Accreditation Council for Graduate Medical Education; IMGs: International medical graduates; LCME: Liaison Committee on Medical Education; LMGs: Lebanese medical graduates

## Competing interests

Kamal F. Badr, MD, is the founding dean of the School of Medicine at the Lebanese American University http://medicine.lau.edu.lb/index.php. The authors have no other potential competing interests to declare.

## Authors' contributions

KFB wrote the first draft the manuscript and EAA contributed to subsequent versions. Both authors read and approved the final manuscript.

## Pre-publication history

The pre-publication history for this paper can be accessed here:

http://www.biomedcentral.com/1472-6963/10/167/prepub

## Supplementary Material

Additional file 1 **Description of Lebanese medical schools**. Describes the characteristics of Lebanese medical schools. Click here for file
