# Supplementary material for: Regionalizing healthcare: a vision for transforming Lebanon into a regional academic hub
Source: BMC Health Serv Res. 2010 Jun 16;10:167. doi: 10.1186/1472-6963-10-167 (PMC2896356; doi:10.1186/1472-6963-10-167)
Supplement: Additional file 1 — Description of Lebanese medical schools. Describes the characteristics of Lebanese medical schools. [file 1472-6963-10-167-S1.DOC]

**Additional file 1:** Description of Lebanese medical schools

|  | **Instruction began** | **Admission requirement** | **Language(s) of instruction** | **Length of studies** | **Degree granted** | **Type** |
| --- | --- | --- | --- | --- | --- | --- |
| American University of Beirut (AUB) | 1868 | Bachelor degree | English | 4 years | Doctor of Medicine (M.D.) | Private |
| Université Saint Joseph (USJ) | 1883 | High school graduation | French | 7 years | Doctor of Medicine (M.D.) | Private |
| Lebanese University (LU) | 1983 | High school graduation | English, French | 7 years | Doctor of Medicine (M.D.) | Public |
| Beirut Arab University (BAU) | 1995 | High school graduation | English | 6 years | Bachelor degree in Medicine and Surgery (M.B.B.Ch.) | Private |
| University of Balamand (UOB) | 2000 | Bachelor degree | English | 4 years | Doctor of Medicine (M.D.) | Private |
| Holy Spirit University of Kaslik (USEK) | 2002 | High school graduation | English, French | 7 years | Doctor of Medicine (M.D.) | Private |
| [Lebanese American University](http://www.lau.edu.lb/) (LAU) | 2009 | Bachelor degree | English | 4 years | Doctor of Medicine (M.D.) | Private |
